# Bilateral Ulnar-Sided Wrist Pain due to Pisiform-Hamate Coalition

**DOI:** 10.1155/2019/5891972

**Published:** 2019-11-27

**Authors:** Ingo Nolte, Flavien Mauler, Tomás Sánchez

**Affiliations:** ^1^Division of Hand Surgery, Kantonsspital Olten, Olten, Switzerland; ^2^Clinic of Hand, Reconstructive and Plastic Surgery, Kantonsspital Aarau, Aarau, Switzerland

## Abstract

Coalition between the pisiform and the hamate is a rare congenital anomaly, often presenting as an asymptomatic incidental finding on radiographs. In some cases, it may become symptomatic, typically after trauma. We present a 13-year-old patient, with no history of trauma, presenting a bilateral painful coalition of the pisiform and hamate. Both of which were treated with excision of the pisiform resulting in complete pain relief. Pisiform-hamate coalition is a rare condition, which can become symptomatic even without any trauma or overuse activity, and is an important differential diagnosis in ulnar-sided wrist pain.

## 1. Introduction

Carpal coalition is a rare congenital anomaly characterized by the union between two or more bones [1]. It is mostly an asymptomatic incidental finding on radiographs, which makes its true incidence unknown [2]. The estimated incidence ranges from 0.1% in Caucasians to 8% in some African ethnies [3, 4]. The most common coalition is found between the lunate and triquetrum [4]. Coalition between the pisiform and the hamate is a rare condition, only described in a few case series and case reports [3, 5–14]. This report highlights the fact that a coalition between the pisiform and hamate is an important differential diagnosis in chronic ulnar-sided wrist pain, and its surgical excision may completely relieve the pain.

## 2. Case Report

**Figure 1:**
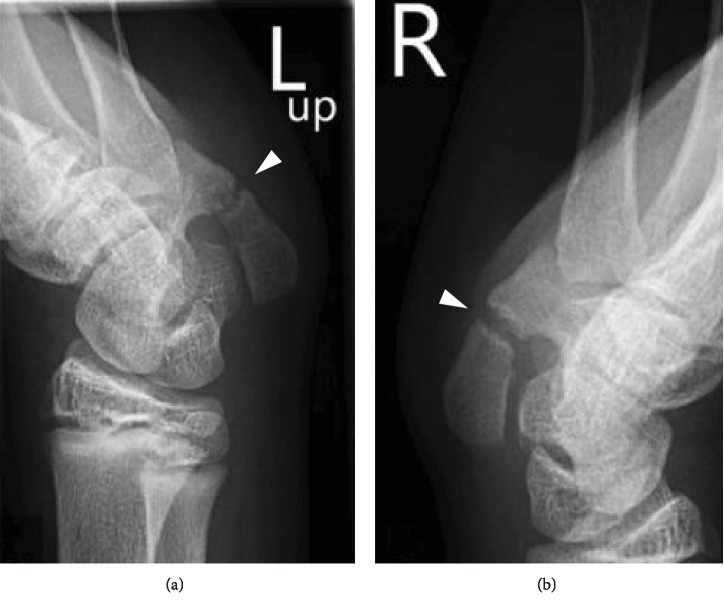
Radiographs of the left wrist (a) and right wrist (b) showing on both sides a coalition between the pisiform and the hook of the hamate (arrowheads).

**Figure 2:**
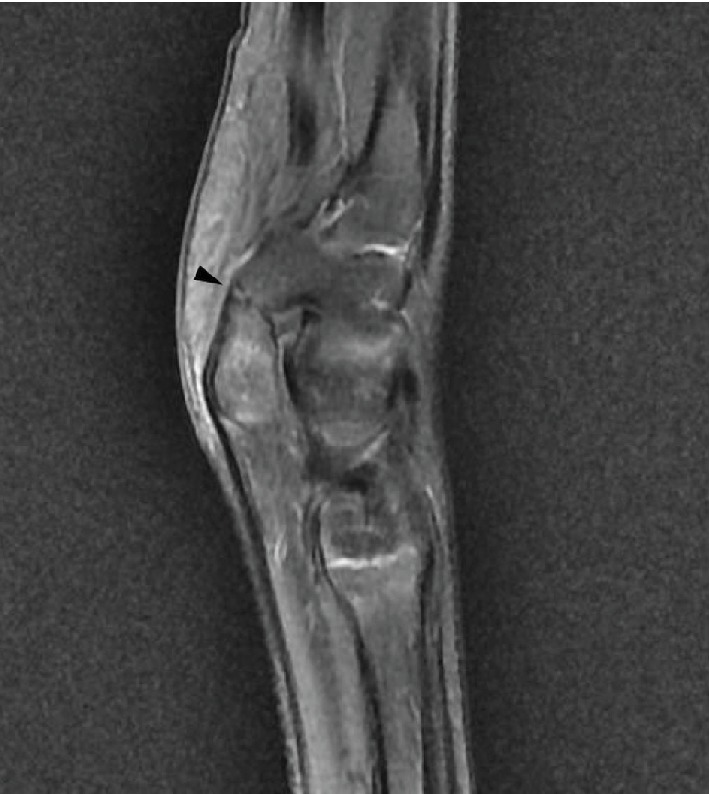
Preoperative MRI of the left wrist showing a synchondrosis coalition of the pisiform with the hook of the hamate (arrowhead).

A 13-year-old, right-hand dominant boy with African origins was referred to our consultation because of bilateral ulnar-sided wrist pain, with no history of trauma. At physical examination, tenderness was elicited by direct palpation of the slightly swollen skin over the pisiform on both sides. Symptoms could be reproduced by extreme ulnar deviation of the wrist. The sensation and motor function were preserved, and Hoffman-Tinel's sign along the ulnar nerve was negative. Radiographs from both sides showed a partial coalition between the pisiform and the hook of the hamate (Figure 1). The diagnosis was confirmed with magnetic resonance imaging (MRI) of the left wrist, which showed a synchondrosis coalition of the pisiform with the hook of the hamate (Figure 2).

Surgical exploration was performed first on the more symptomatic left-hand side. Guyon's canal was opened and explored through a longitudinal incision. The ulnar artery and nerve were mobilized and retracted radially. The synchondrosis was exposed, and the pisiform was detached from the flexor carpi ulnaris tendon. The pisiform was removed entirely, and the remaining hook of the hamate was rounded with a rongeur. The wrist was then immobilized in a plaster cast for two weeks postoperatively. Nine weeks after the surgery, the patient reported to be pain free, and the other side was operated in the same way. At the last visit, one year postoperatively, the patient was pain free on both sides with a grip strength of 39 kg on the left side and 40 kg on the right side, and a wrist motion of 75° of flexion and 70° of extension on the left side and 75° flexion and 75° extension on the right side.

## 3. Discussion

Coalition of two or more carpal bones is a congenital anomaly, usually occurring between carpals of the same row (longitudinal type) with lunate-triquetrum coalition being the most common one [1]. Hamate-pisiform coalition represents a coalition between a carpal bone of the proximal and the distal row (transversal type) and is a rare condition with a frequency estimated between 0.11% and 0.76% [1]. Coalition between the pisiform and hamate was first described in the English literature in 1959 by Cockshott [15]. The main reason for carpal coalition is thought to be a failure of segmentation of the common cartilaginous precursor of the intercarpal joints during the fourth and eighth weeks of embryonic life [16]. However, this mechanism does not apply for the pisiform and the hamate as they are not formed from a cartilaginous joint. Cockshott suggested an ossification of the distal portion of the flexor carpi ulnaris; other authors have hypothesized metaplasia of the pisohamate ligament into the bone [1–4, 15].

The present case is a type 1 coalition according to the classification of Devilliers Minnaar [17], showing an incomplete fusion resembling a pseudoarthrosis (fibrocartilage coalition). Type 2 would have shown an incomplete osseous fusion, type 3 a complete fusion, and type 4 a complete fusion with associated anomalies. All reported painful cases were either type 1 or type 2 [7]. Our patient presented a bilateral coalition between the pisiform and hamate, which became symptomatic on both sides without history of trauma or repetitive use. As far as we know, this makes this case unique in the English literature since most reports relate to some form of physical stress or trauma and are mostly unilateral.

The removal of the pisiform which is not a very demanding procedure, commonly used for pisotriquetral osteoarthritis, was able to completely resolve the symptoms on both sides. This is why we would recommend this as the treatment of choice if facing this condition.

## 4. Conclusion

Coalition between the pisiform and hamate is a rare congenital condition which is mostly an asymptomatic incidental finding. However, it can become painful with or without any history of trauma and is an important differential diagnosis in chronic ulnar-sided wrist pain. Surgical excision of the pisiform may completely relieve the pain.
